# Approximating deformation fields for the analysis of continuous heterogeneity of biological macromolecules by 3D Zernike polynomials

**DOI:** 10.1107/S2052252521008903

**Published:** 2021-10-14

**Authors:** David Herreros, Roy R. Lederman, James Krieger, Amaya Jiménez-Moreno, Marta Martínez, David Myška, David Strelak, Jiri Filipovic, Ivet Bahar, Jose Maria Carazo, Carlos Oscar S. Sanchez

**Affiliations:** a Centro Nacional de Biotecnologia-CSIC, C/ Darwin 3, Cantoblanco, Madrid 28049, Spain; b Department of Statistics and Data Science, Yale University, New Haven, Connecticut, USA; c Department of Computational and Systems Biology, University of Pittsburgh, Pennsylvania, USA; d Institute of Computer Science, Masaryk University, Botanická 68a, 60200 Brno, Czech Republic; e Faculty of Informatics, Masaryk University, Botanická 68a, 60200 Brno, Czech Republic

**Keywords:** multi-dimensional scaling (MDS), 3D reconstruction and image processing, single-particle cryo-EM, spherical harmonics, Zernike polynomials, conformations

## Abstract

Structural biology has evolved greatly due to the advances introduced in fields like electron microscopy. This image-capturing technique, combined with improved algorithms and current data processing software, allows the recovery of different conformational states of a macromolecule, opening new possibilities for the study of its flexibility and dynamic events. However, the ensemble analysis of these different conformations, and in particular their placement into a common variable space in which the differences and similarities can be easily recognized, is not an easy matter. To simplify the analysis of continuous heterogeneity data, this work proposes a new automatic algorithm that relies on a mathematical basis defined over the sphere to estimate the deformation fields describing conformational transitions among different structures. Thanks to the approximation of these deformation fields, it is possible to describe the forces acting on the molecules due to the presence of different motions. It is also possible to represent and compare several structures in a low-dimensional mapping, which summarizes the structural characteristics of different states. All these analyses are integrated into a common framework, providing the user with the ability to combine them seamlessly. In addition, this new approach is a significant step forward compared with principal component analysis and normal mode analysis of cryo-electron microscopy maps, avoiding the need to select components or modes and producing localized analysis.

## Abstract 2

A new tool based on 3D Zernike polynomials is presented that allows the study of the continuous heterogeneity of biological macromolecules, revealing the structural relationships present among different states by the approximation of deformation fields.

## 1. Introduction   

The application in electron microscopy of techniques such as cryo-electron microscopy (cryo-EM), single-particle analysis (SPA) (Carroni & Saibil, 2016 ▸) or electron cryo-tomography (Schur, 2019 ▸) has proven to be a versatile tool to trace high-resolution structures. In particular, cryo-EM SPA has proven to be especially good at providing not only one structure, but a series of them, with most methods aiming to resolve stable states that are referred to as classes. In this way, we get a first approximation to the conformational landscape of the macromolecule, albeit restricted to these stable states.

However, the limited number of classes that can be extracted from a 3D classification is usually not enough to unveil fully the dynamics of a given macromolecule. The complete characterization of a conformational landscape can only be achieved through the analysis of multiple transient and stable states needed to describe the molecular flexibility in a more accurate manner. The knowledge of these transient and stable states leads to a better description of how structural changes might affect molecular function or interaction affinity, among other properties of interest.

The formulation we introduce here is oriented towards modelling continuous flexibility (Sorzano *et al.*, 2019 ▸), which can be used to characterize the motions undergone by a molecule when exploring different states. We have already addressed this problem in our previous work on continuous heterogeneity using normal mode analysis (NMA) (Sanchez Sorzano *et al.*, 2016 ▸). However, this process relied on manual selection of the modes describing the structural changes reflected by two cryo-EM maps, thus making the analysis of molecular flexibility more complex for the user. The new algorithm that we propose in this work tries to address this problem by simplifying the analysis for the user.

In our new methodology, there is no longer a normal modes space where some choices have to be made. Instead, a totally new approach is presented here, based on an expansion on a 3D basis that does not require user intervention at all. We have also improved the analysis of pairwise comparisons by introducing a multidimensional scaling algorithm that automatically combines the outputs from two different metrics. Finally, the new algorithm also allows the analysis of local strains and rotations, as done by us earlier (Sorzano *et al.*, 2016 ▸), with the advantage of having all the analyses integrated into a single mathematical framework. We provide a more in-depth comparison with alternative methods in Section 2.1.

The paper makes the following major contributions.

(i) The development of an automatic algorithm to analyse continuous heterogeneity of macromolecules through cryo-EM maps.

(ii) Representation of the strain and rotation components defining a transition between two different conformational states.

(iii) Representation of a series of conformations in a structure mapping and consensus of different mappings defined by different comparison metrics.

(iv) A methodology to compare cryo-EM maps with simulated data.

(v) The application of deformation fields to atomic structures to predict different conformations given by a series of cryo-EM maps.

## 2. Methods   

### 2.1. Determining structural deformations   

In order to detect the movements defining a conformational transition between two states of the same macromolecule, we need to determine the displacements that each region of the molecule will undergo between the two states. The key development in this work is the successful expression of the maps in terms of a mathematical basis on which the displace­ments are calculated. Although full details are provided in Appendices *A* [App appa] and *B* [App appb], here it suffices to say that we use a generalized form of Zernike polynomials to expand functions on a ball (as the macromolecule we are interested in is defined inside a spherical volume). This is not the only possible choice of basis functions [for example, it would have been possible to use the Laguerre polynomials described by Provencher & Voguel (2010 ▸) or the prolate spheroidal functions (Greengard & Serkh, 2018 ▸)], and we do not expect superiority of any of these possible bases as long as all of them are bases of functions defined within a sphere. Additionally, we find that Zernike polynomials have some appealing mathematical properties especially well suited to our problem. Indeed, these Zernike polynomials allow for the expansion of functions on a sphere which do not vanish at the boundaries (so that the more external parts of the macromolecule can move). Moreover, the basis is closed under rotations. In Appendix *B* [App appb] we further explore its properties and its relationship to spherical harmonics.

Considering a pair of electron-density maps representing two conformational states of a macromolecule, it is possible to pose the displacement-finding problem as

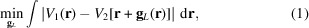

where *V*_1_ and *V*_2_ represent two conformations of a given molecule. Here it is important to note that we are measuring the distance between the target and the distorted volumes in terms of the *L*_1_ norm. Although it would also have been possible to use the *L*_2_ norm, we have chosen this definition as it is more robust to outliers (*i.e.* it is more robust to those cases where the maps do not match completely or have missing regions). The displacement to be applied to the coordinates of *V*_1_ is defined by the deformation field **g**(**r**) parameterized through the expansion in Zernike polynomials *Z*_*l*, *n*, *m*_(**r**) (see Appendix *A* [App appa]),

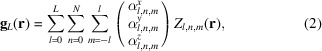

where *N* and *L* represent the maximum allowed degrees for the Zernike polynomials and the corresponding spherical harmonics, respectively.

The amount of displacement at every point is controlled by the deformation coefficients **α**_*l*, *n*, *m*_. Our objective is to find the deformation coefficients that minimize the goal function in equation (1). This is achieved through a Powell’s conjugate direction method starting from an initial guess of **α**_*l*, *n*, *m*_ = 0 for all indices *l*, *n*, *m* and directions *x*, *y*, *z* (that is, no deformation). This initialization of the minimization method assumes that the identity/equilibrium solution (**α**_*l*, *n*, *m*_ = 0) is close enough to the real solution defining the structural transition represented by the cryo-EM maps. Since in most of the cases this assumption is fulfilled, this initial guess allows the minimization method to find the set of coefficients that appropriately describes the motion between the two maps. However, it is important to note that there are many local minima where the minimization process could be trapped. In this respect, and although in our experience the initialization conditions proposed in this work provide results close enough to the ideal solution, there could be cases in which other ways to initialize the algorithm could be more beneficial in terms of minima search.

The deformation field estimated above can be submitted to the local strain and rotation analysis described by Sorzano *et al.* (2016 ▸). This analysis reveals the nature (stretching, compression or rotation) of the local forces acting on *V*_1_ to transform it into *V*_2_ as well as their local intensity.

In our deformation model, it is possible to divide the movements that a molecule may undergo into ‘low’- and ‘high’-frequency movements, depending on how localized these movements are, *e.g.* a transition from an open to a closed state can be considered a low-frequency movement, while the rotation of a specific α-helix might be a high-frequency movement. Parameters *L* and *N* specify the maximum degree of the polynomials used in the description of molecular flexibility. In this way, we may control the maximum frequency of the movements that could be analysed by the basis. Obviously, analysing larger *L* and *N* will result in a longer computational time, because more **α**_*l*, *n*, *m*_ coefficients will need to be determined and there is a higher risk of overfitting. However, the larger the values given to the parameters *L* and *N*, the higher the frequencies the algorithm will be able to analyse (although in general, global motions dominate the conformational change; Bahar *et al.*, 2010 ▸).

Although in many cases analysing global motions is enough to describe in a precise manner the structural changes a macromolecule may undergo, it can be the case that the motions of interest are focused on a very localized area of the molecule. In that case, being able to go to higher degrees on the basis will allow the algorithm to study those motions specifically, without modifying the areas that should remain still. Another possibility is direct restriction of the structural analysis to any specific region in the macromolecule by centring a sphere on that area and selecting an appropriate radius. In this way, it will not be necessary to reach very high degrees in the basis (thus reducing the computational complexity). However, by imposing these kinds of restrictions the algorithm might include artefacts in the surface of the sphere as the molecular regions outside of it will remain untouched. Depending on the molecule and motions to be analysed, the researcher can decide which analysis will be more appropriate for a specific case.

It is important to mention that only in a very few cases did we need to increase the degree of the basis to analyse a localized motion that we were interested in, or have to play with the regularization parameter to get a better approximation of the deformation fields, since the default values were good enough for most of the experiments we have performed so far.

To reduce the possibility of overfitting as much as possible, we regularize the cost function by adding two penalty terms, 

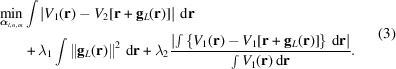

The first term of the regularization penalizes excessive deformation and the second penalizes changes in the mass of *V*_1_ due to the deformation. Regularization terms λ_1_ and λ_2_ are usually given low values to prevent large deviations from the ideal solution. Nevertheless, both can be set by the user to any value they consider appropriate for their specific analysis. The guideline for their selection should be that the three terms in the goal function should have values of the same order of magnitude. In our implementation, we report the three contributions helping the user to choose these multipliers.

### 2.2. Relationship to other continuous deformation models   

Probably the two most widely continuous deformation models used by the structural biology community in mapping the conformational space of biomolecules (or in analysing cryo-EM images) are principal component analysis (PCA) (Tagare *et al.*, 2015 ▸) and normal mode analysis (NMA) (Cui & Bahar, 2006 ▸). The three models (PCA, NMA and 3D Zernike) claim to be bases for continuous movements. However, as will be clarified below, they define bases of different mathematical entities.

PCA considers a volume of size *N* ^3^ voxels as a vector in 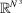 . Due to the continuous heterogeneity and the uncertainty in the 3D reconstruction process, the reconstructed map can be considered as the mean of a set of other vectors (maps) whose projections are acquired by the microscope. If we consider the covariance matrix associated with that set of maps (a matrix of size *N* ^3^ × *N* ^3^), then the principal components form a basis (if the covariance matrix is not degenerate) in which the set of maps can be linearly expressed. The PCA approach approximates the deformed volume by a linear combination of volumes (the principal directions), 

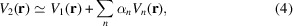

where *V*_*n*_ are the eigenvolumes of the PCA decomposition. The undeformed model is then obtained by subtracting the appropriate amount of each of the eigenvolumes, 

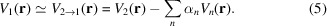

Due to the low-frequency nature of the PCA principal directions (Sorzano & Carazo, 2021 ▸), the undeformed volume is necessarily of low resolution.

In our model, we assume that any deformed volume *V*_2_ can be undeformed by applying **g**_*L*_,

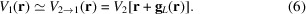

Zernike polynomials provide a basis for **g**_*L*_(**r**), not the volumes. Our model revolves around the location of the voxel (which implies a nonlinear relationship between *V*_1_ and *V*_2_), providing an intrinsically better handling of the local characteristics of the map, while in PCA there is a linear model at the level of the volumes themselves (not their internal co­ordinates).

Our approach has another potential advantage over the PCA model: it can easily be applied to atomic structures fitted into *V*_1_. For any given atom in the atomic structure at a position **r**_1_, that is defined in the same coordinate system as *V*_1_, we simply have to move it to the location **r**_1_ + **g**_*L*_(**r**_1_).

In NMA, volumes are approximated by a set of *P* pseudo­atoms with weights *c*_*p*_ and basis function *b*(**r**) located at the locations **r**_*p*_ (Jonić & Sorzano, 2016 ▸), 

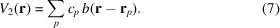

NMA is based on a second-order Taylor approximation of the energy landscape of the macromolecule, starting with a description of the interactions between the pseudoatoms. This is typically treated using an elastic network model where pseudoatoms within a distance criterion are connected by harmonic springs (Bahar *et al.*, 2010 ▸). The associated Hessian is of size 3*P* × 3*P* and the normal modes are its eigenvectors (sorted by increasing eigenvalue) and a basis of the 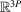 space. Let us call 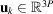 the *k*th normal mode, and 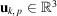 the part of the normal mode corresponding to the *p*th pseudo­atom. To deform *V*_2_ to make it similar to *V*_1_ we consider the first *K* normal modes with different weights α_*k*_, 

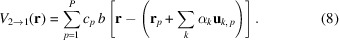

Similar to our method, NMA acts by displacing the location of the pseudoatoms (our model acts by displacing the location at which we must interpolate *V*_2_). However, an advantage of our new method with respect to NMA is that the NMA deformation is only known at the location of the pseudoatoms, while our new method is fully defined within the sphere containing the macromolecule. In this way, the NMA would be a discretized version of the underlying continuous deformation field, while 3D Zernike polynomials would be an estimate of that continuous field.

Summarizing, each of the methods described so far (PCA, NMA and 3D Zernike polynomials) has a basis in different mathematical entities (vectors in 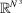 , 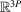 or the set of square integrable functions defined within the sphere of a given radius). 3D Zernike polynomials have the advantage that they are defined for every point in the macromolecule (as opposed to NMA) and the undeformed volumes do not lose resolution (as opposed to PCA).

Elastic deformations have also become popular for the alignment of frames within a movie (Abrishami *et al.*, 2015 ▸; Tegunov & Cramer, 2019 ▸; Zheng *et al.*, 2017 ▸). Although they have not been explicitly used to deform volumes, one could envision that they could be easily extended to three dimensions. This would certainly be a possible approach and we earlier used cubic splines for this purpose (Sorzano *et al.*, 2016 ▸). However, the basis used in this paper, which is defined exclusively within a sphere, is more appropriate for the task at hand (describing a function whose support is fully contained within that sphere) than for a more generic set of functions that constitute a basis of functions defined within a cube. This ‘greater appropriateness’ translates into requiring fewer coefficients to express the same deformation field to the same level of accuracy.

### 2.3. Distances between a set of maps   

In most practical cases, the number of states that can be reconstructed by cryo-EM SPA is larger than two, which naturally implies the generalization of the case presented above to a number of pairwise operations capturing the different structural relationships among the set of maps under consideration. This information is summarized in a graph known as a structure map (Sanchez Sorzano *et al.*, 2016 ▸) or conformational landscape (Zhang *et al.*, 2021*b* ▸), which represents each conformation as a point in conformational space. The closer two points are in the structure map, the more similar they are.

By estimating the Zernike polynomial deformation for all possible pair combinations in a set of *N* cryo-EM maps, a distance matrix can be computed in which we measure how far two cryo-EM maps are from each other. The deformation field between the two cryo-EM maps **g**_*L*_(**r**) provides a mechanism for calculating such a distance. For instance, we may define the distance between two cryo-EM maps *V*_1_ and *V*_2_ as the sum of the lengths of the deformations at each point, 

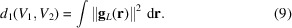

Besides equation (9), there are additional sensible ways of defining the distance between two cryo-EM maps. One of them consists of measuring the correlation between *V*_1_ and *V*_2_ once *V*_2_ is undeformed to resemble *V*_1_, 

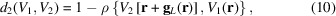

where ρ is Pearson’s correlation coefficient.

By comparing all cryo-EM maps, we would construct a matrix of the distances of all versus all maps.

It is worth mentioning here that, in order to get accurate comparison measurements, it is desirable to have a set of cryo-EM maps with similar characteristics. In particular, it is important to filter the maps in the set so that all their resolutions match the lowest value present in the data set. In this way, the structure mappings and distance matrices will not be affected by resolution changes, leading to a more meaningful projection of the different maps in the low-dimensional space resulting from the application of this method.

### 2.4. Embedding of conformations using multiple multidimensional scaling   

Once we have the above-mentioned distance matrix, we may use multidimensional scaling (MDS) (Härdle & Simar, 2012 ▸) to find points in a low-dimensional space of dimension *p* (typically *p* = 2 or *p* = 3 for ease of representation) such that the distances between points in the low-dimensional space represent in some form the distances between the cryo-EM maps in the full dimensionality space [*e.g.* equation (9)]. For a detailed description of MDS, see Härdle & Simar (2012 ▸). If we have *N* cryo-EM maps to compare, let us refer to the matrix collecting all the points in the low-dimensional space as *X*_1_ [ 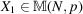 , that is, the set of cryo-EM maps of size *N* × *p*]. The subscript 1 indicates that we used *d*_1_ to perform the low-dimensional mapping.

If instead of equation (9) we use equation (10), then this would give us another MDS representation *X*_2_. While the distance *d*_1_ concentrates on the amount of deformation required to transform *V*_1_ into *V*_2_, *d*_2_ describes the distance between *V*_1_ and *V*_2_ after applying the inverse deformation to *V*_2_.

We could similarly conceive other strategies to measure the distance between any pair of cryo-EM maps *V*_1_ and *V*_2_. None of them should necessarily be better than the others, since each one addresses the problem from a different perspective. In this regard, it is impossible to favour any one of the different metrics without a specific task to accomplish. However, it is still sensible to combine the different mappings induced by each one of the distances as a way of producing a single summary of all their information. For the task of producing such a summary, we propose to construct a combination of the embeddings that minimizes the entropy of the result, understanding that the entropy is reduced when more order is found.

At this point and following the aforementioned idea, we may want to combine all those low-dimensional mappings into a single set of points to summarize the relative distances derived from each distance definition. For doing so we have found useful the following procedure that we call multiple multidimensional scaling:

(i) We take one of the mappings as reference, for instance, *X*_1_.

(ii) We look for the affine transformation *T*_*i*_ that minimizes the Frobenius norm [for an arbitrary matrix *A*, its Frobenius norm is defined as 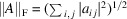 ] between each *X*_*i*_ transformed mapping and the reference mapping (since the MDS mappings of different distances, performed in an independent way, normally result in mappings of different scales, central locations, rotations and mirrors),

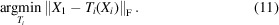

For convenience of notation, let us define *T*_1_(*X*_1_) = *X*_1_.

(iii) The consensus mapping is constructed as the convex combination of all transformed mappings (the determination of the specific α_*i*_ coefficients for the combination will be addressed in the following step), 

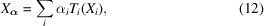

with the constraints α_*i*_ ≥ 0 and 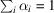 (with these constraints *X*_**α**_ is said to be a convex combination of the input matrices). Note that the *j*th row of the matrix *X*_**α**_ (referred to as **x**_*j*, **α**_) indicates the position of the *j*th cryo-EM map in the low-dimensional space (whose dimension is *p*). For each one of the consensus candidates we associate the probability density function 

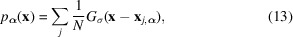

where *G*_σ_ is a *p*-multivariate spherical Gaussian whose covariance matrix is σ^2^ *I* {in our experiments, we chose 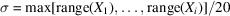 , where range(*X*_*i*_) is the difference between the maximum and minimum values of any of the components of the mapped vectors}.

(iv) Since the best combination of coefficients α_*i*_ is not known beforehand, each possible convex combination has to be analysed. The criterion followed was to look for the convex combination that minimized the Shannon entropy of the probability density function defined above, 

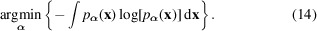

The rationale is that we are looking for the convex combination that brings maximum order to the low-dimensional mapping.

We observe that the procedure described above normally finds a good balance between the properties of the different low-dimensional mappings, resulting in well structured summaries.

## 3. Results   

This algorithm has been implemented in *Xmipp* (de la Rosa-Trevín *et al.*, 2013 ▸) and it is available through *Scipion* (de la Rosa-Trevín *et al.*, 2016 ▸) under the protocols named volume deform - Zernike3D and struct map - Zernike3D.

We performed some tests with a pair of maps to compare these two implementations to analyse the performance improvement. The maps used for the tests had dimensions of 250 in *X*, *Y* and *Z*, leading to averaged execution times of 1 h and 20 min (CPU) and 39.5 s (GPU). The tests were performed with an Intel i7-9750H and a Nvidia 2060 with Cuda 10.1, respectively.

### 3.1. Experiment 1: cryo-EM maps of the human mitochondrial ribosome   

**Figure 1:**
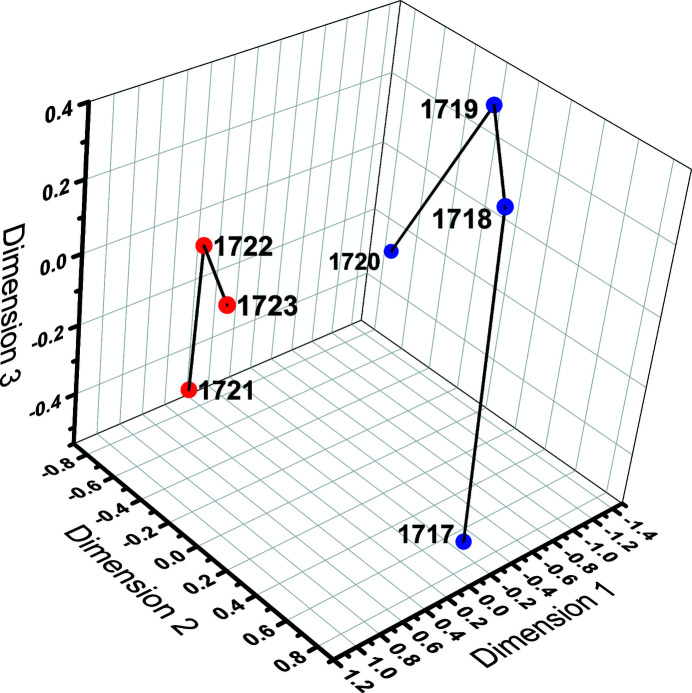
Structure mapping recovered a set of seven maps of the human mitochondrial ribosomes (Amunts *et al.*, 2015 ▸) from the data set retrieved from the EMDB after running the *Zernike3D* algorithm. Two trajectories are suggested that might correspond to two independent states (pre-translocation and post-translocation) present in the data set, consistent with results from a previous normal-mode-based structure mapping algorithm (Sanchez Sorzano *et al.*, 2016 ▸). The labels refer to the EMDB entries.

We first tested our approach using a small data set covering a range of conformational states of a human mitochondrial ribosome (Amunts *et al.*, 2015 ▸), as previously described by Sanchez Sorzano *et al.* (2016 ▸). To check whether the structure map suggested two independent (pre-translocation and post-translocation) states following different conformational transitions as found in our previous study (Sanchez Sorzano *et al.*, 2016 ▸), we applied the methodology described above with *N* = 3 and *L* = 2 (the maximum allowed degrees for the Zernike polynomials and the spherical harmonics, respectively). As expected, the structure map indeed suggests two independent arrangements following their own conformational transitions, grouped as red and blue dots in Fig. 1 ▸ (the black line segments joining the dots are just provided to enhance visualization). We thus conclude that the new approach is capable of reproducing the results of previous supervised methods that perform similar analyses and accurately groups the seven cryo-EM structures [indicated by their EMDB (Electron Microscopy Data Bank, https://www.ebi.ac.uk/emdb/) identification numbers] into two groups of conformers, each representative of a different functional state.

**Figure 2:**
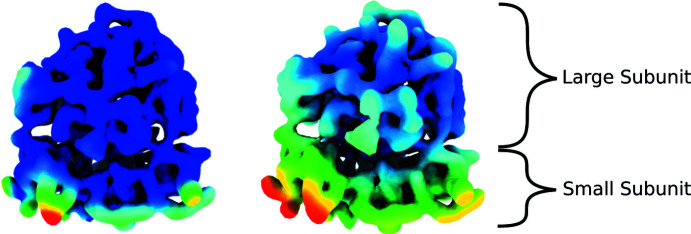
Mitochondrial ribosome subunits 28S and 39S (from EMDB entry 1720) coloured using the strain (left) and rotation (right) components extracted from the deformation coefficients obtained when analysing the motion described by EMDB 1720 and EMDB 1723. The conformational change described by these two maps is represented in Video 1.

Additionally, the new approach also allows for the local decomposition of the deformation field into local strains and local rotations, as was done by Sorzano *et al.* (2016 ▸). The representation of these two components is shown in Fig. 2 ▸ for one of the pairs of ribosomes (EMDB entries 1720 and 1723). In addition, Video 1 in the supporting information shows the conformational changes described by these two maps. For this video, we coloured the ribosomes using the rotation component represented in Fig. 2 ▸ to simplify their comparison. According to this analysis, the rotations appear to be distributed through the whole structure of the ribosome, although the larger rotations (shown in red) are mostly found in the small subunit. Similarly, the strains are mainly localized in the small subunit and appear to be less distributed. This reveals that the basis is capable of deforming in a localized fashion, leading to a better description and identification of the different movements that define the transition between the two conformations. It is also possible to see that we are obtaining results comparable with those found by Sorzano *et al.* (2016 ▸), with the advantage of having all these analyses unified in the same framework, which implies an overall simplification leading to more complete studies.

### 3.2. Experiment 2: trajectory recovery of the CCT complex   

Our next experiment is aimed at characterizing the ability of the method to recover the sequence of events present in a set of conformations defining a certain trajectory in conformational space. Such conformations can be created computationally by taking advantage of biophysical methods such as molecular dynamics simulations (MD) (Adcock & McCammon, 2006 ▸) and normal mode analysis (NMA) (Bahar *et al.*, 2010 ▸), simulating the movements defining a transition between two conformations. In this case, we used a trajectory from a recent study (Zhang *et al.*, 2021*b* ▸), which was generated using a purely NMA-based approach called the adaptive anisotropic network model (adaptive ANM; Yang *et al.*, 2010 ▸) implemented in *ProDy* (Zhang *et al.*, 2021*a* ▸). This gave us 30 different models along an open–closed transition of the mammalian chaperonin CCT complex between two atomic models derived from a previous cryo-EM study (Cong *et al.*, 2012 ▸), taken from the Protein Data Bank (PDB) (wwPDB Consortium, 2019 ▸), as described by Zhang *et al.* (2021*b* ▸). The starting structure with one ring open and one ring closed (PDB entry 4a0w) (Cong *et al.*, 2012 ▸) corresponded to an ATP-bound state and the target structure with both rings in an intermediate conformation (PDB entry 4a13) (Cong *et al.*, 2012 ▸) corresponded to the ADP-bound state, allowing us to explore the conformational changes triggered by ATP hydrolysis. In the adaptive ANM method, all steps are based on coarse-grained normal modes calculated using the anisotropic network model (ANM) (Atilgan *et al.*, 2001 ▸; Doruker *et al.*, 2000 ▸; Eyal *et al.*, 2006 ▸), providing coordinate changes for C^α^ atoms only. At each step, normal modes were selected that had the highest directional overlap (correlation cosine) with the deformation vector between the current conformation and the target structure up until the sum of the squared overlaps exceeded a threshold of 0.4. The contribution of each mode to the deformation was chosen so as to take 20% of the maximum provided by the unnormalized dot products (a scaling factor of 0.2) so as to avoid unphysical deformations while maintaining efficiency. The normal modes were re­calculated until the root-mean-square deviation (r.m.s.d.) from the target structure fell below 1 Å, resulting in a total of 30 steps. Each step recruited a larger number of modes and had a smaller total size as the required deformation became less cooperative and more local (see Video 2). We focus our discussion on the ring that goes from open to intermediate–closed for simplicity.

**Figure 3:**
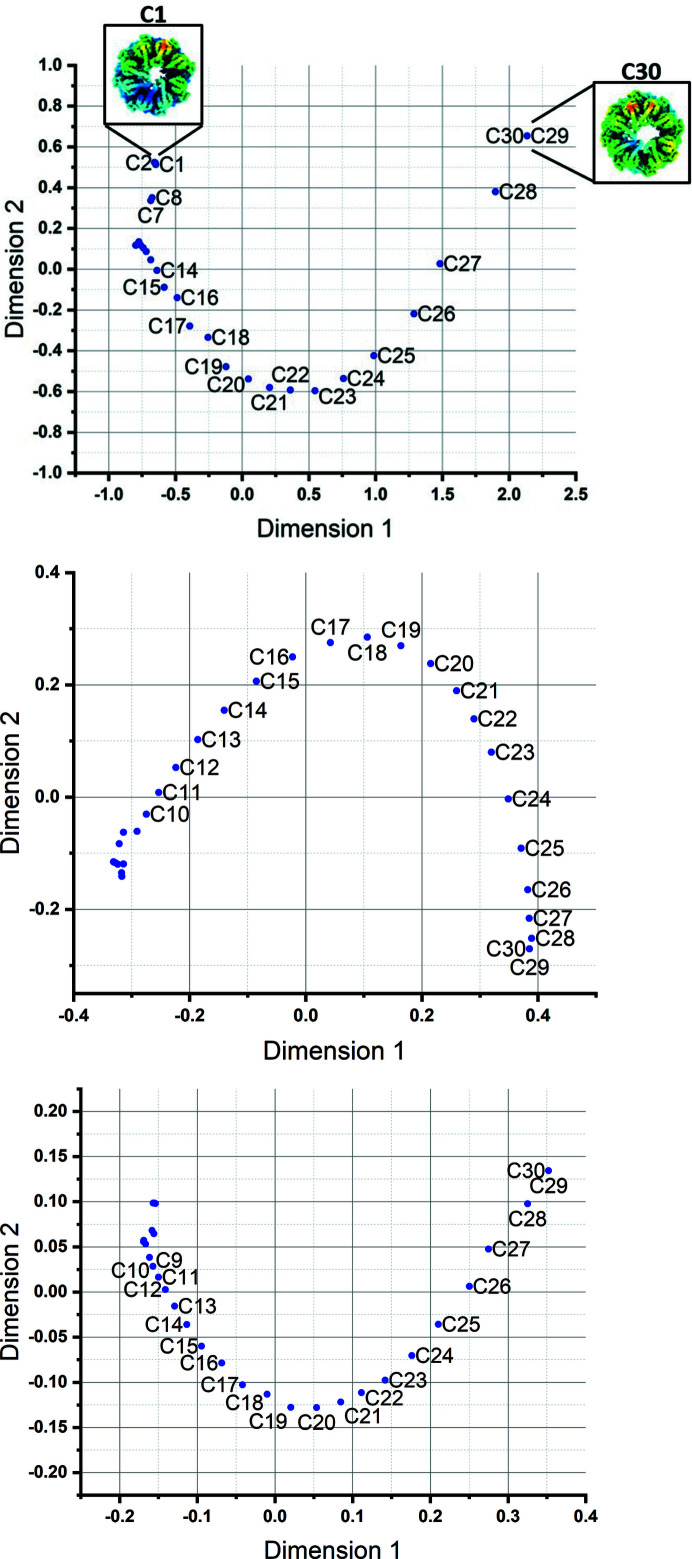
Structure maps of a set of 30 models obtained by an NMA-based approach called adaptive ANM over an open–closed transition of the chaperonin CCT from our previous study (Zhang *et al.*, 2021*b* ▸) using (top) the deformation distance *d*_1_, (middle) the correlation distance *d*_2_ and (bottom) the minimum entropy consensus followed by an MDS analysis of the corresponding distance matrices. The open conformation is labelled as C30 and the closed one is C1. The intermediates predicted along low-frequency modes starting from the open state are labelled C29, C28 *etc.*, whereas the vicinity of C1 populates conformers reached by high-frequency modes. The latter is relatively more sensitive to the metric used in the *Zernike3D*-based evaluation (compare *d*_1_ in the top panel and *d*_2_ in the middle panel). The consensus path (bottom) provides an optimal solution based on the convex combination of the structure mappings shown in the top and middle plots in such a way that the entropy of the final mapping is minimized.

We then transformed these atomic structures into Coulomb potential maps using the electron atomic scattering factors (EASFs) as described in previous work (Sorzano *et al.*, 2015 ▸). Fig. 3 ▸ shows the structure maps recovered after applying our methodology. We can see that the sequential order of the 30 intermediate conformers along the trajectory was successfully recovered by our approach. The direction, however, is arbitrary and in this case the start of the trajectory was numbered as conformer 30 and the end as conformer 1.

With this example, we additionally illustrate the distinct MDS mappings obtained when the distances *d*_1_ [amount of deformation, Fig. 3 ▸ (top)] and *d*_2_ [similarity after deformation, Fig. 3 ▸ (middle)] are used. Although the trajectory was successfully recovered by both distances, the correlation distance *d*_2_ was slightly more accurate in this case. The reason is that most of the changes between the structures at the end of the transition (labelled 1 to 13 by the algorithm) are high-frequency movements (*i.e.* movements of loops or small α-helices and β-sheets) that cannot be fully captured by the Zernike 3D basis with *N* = 3 and *L* = 2 (although larger *N* and *L* would allow one to express these small-detail movements, they would also increase the computational cost). Fortunately, the consensus mapping [Fig. 3 ▸ (bottom)] is able to identify the existence of high-frequency movements and gives more weight to the *d*_2_ mapping (correlation distance) automatically, resulting in an almost exact recovery of the volume sequence along the trajectory.

At least in this case we can conclude that *d*_1_ is very good for describing the low-frequency movements (*e.g.* C23–C30), while *d*_2_ is very good for characterizing the high-frequency differences (*ca* C1–C13), and both perform well in the intermediate-frequency regime. Depending on whether our set of input maps are related by large or small movements, one distance or the other will be better suited to capturing the overall set of relationships. The consensus mapping will thus analyse both mappings and automatically determine the optimal weight that results in a low-dimensional mapping that can be readily interpreted.

### 3.3. Experiment 3: comparison of atomic models and cryo-EM maps from the rabbit ryanodine receptor RyR1   

In the following example, we explored the possibility of matching (pseudo/simulated) cryo-EM maps derived from atomic models with experimental electron microscopy maps in the same low-dimensional space. For this purpose we selected five experimental cryo-EM maps deposited for the ryanodine receptor 1 (RyR1) from rabbit (EMDB entries 8379, 8385, 8390, 8395 and 8373) and their respective atomic models in the PDB (PDB entries 5tam, 5tau, 5taz, 5tb4 and 5t9n).

First, we converted the atomic models into density maps using EASFs, as described in the previous section. Then, to make the cryo-EM maps and atomic models comparable, we also filtered all volumes in the analysis to a common resolution (specifically, to the lowest of the reported resolutions of the cryo-EM maps). Note that without applying this low-pass filter the minimization process of equation (1) might not reach a meaningful minimum. Finally, we applied the method presented in this work to this combined data set.

**Figure 4:**
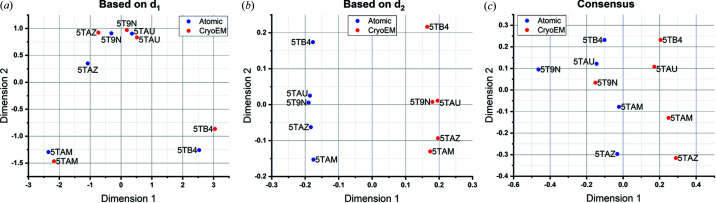
Results obtained after applying the *Zernike3D* algorithm to a set of cryo-EM maps from the ryanodine receptor 1 (RyR1). The data set was constructed in such a way that there are always two maps corresponding to the same conformational state: an experimental cryo-EM map and a cryo-EM map simulated from the atomic structure associated with the previous experimental map. (*a*) A structure map obtained when comparing experimental cryo-EM maps (red dots) and atomic models (blue dots) for RyR1 through the deformation distance *d*_1_. The results show that the method succeeded in recovering most of the pairs defined by the experimental cryo-EM maps and atomic structures. (*b*) A structure map obtained when comparing experimental cryo-EM maps (red dots) and atomic models (blue dots) through the correlation distance. In this case, the correlation metric fails to recover the pairs but it identifies correctly the two different map types used for this analysis. (*c*) A consensus structure map resulting from the combination of (*a*) and (*b*). The consensus provides an optimal solution that helps to identify the map pairs and the map types by keeping a similar structural relationship in the blue and red branches. In these cases, none of these approaches are sufficient for creating a meaningful structure map based on conformation alone, leading us to apply the improvement in Fig. 5 ▸.

**Figure 5:**
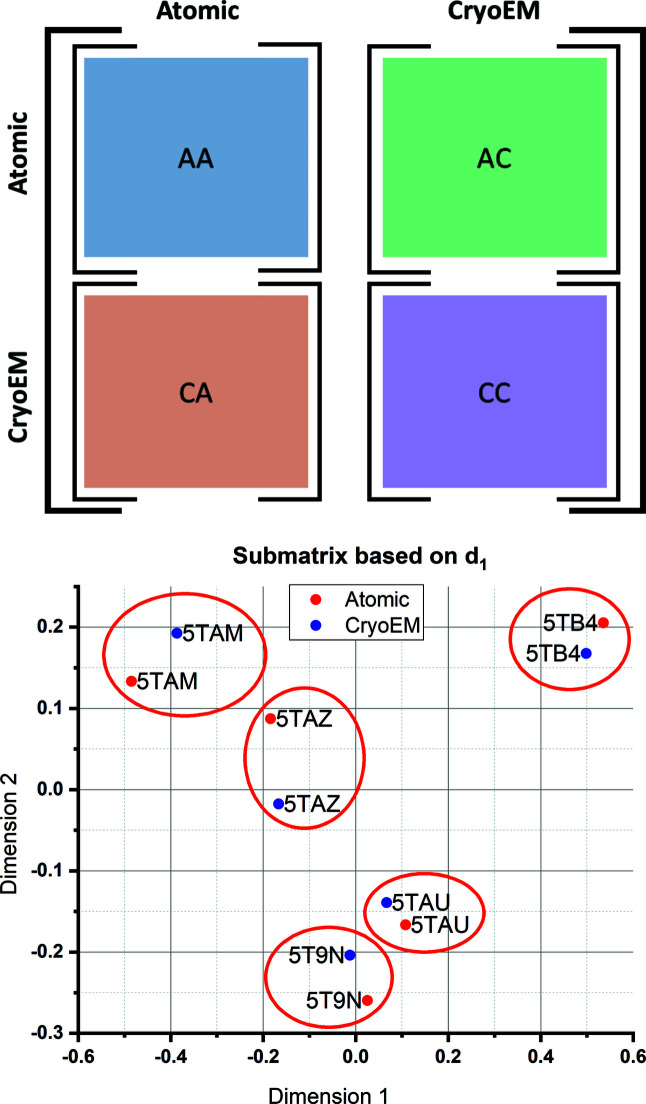
Results obtained after applying the *Zernike3D* algorithm to a set of cryo-EM maps from the ryanodine receptor 1 (RyR1) followed by a decomposition of the distance matrix computed by the algorithm into different blocks to recover more meaningful structure mappings. (Top) A partition of the distance matrix into 2 × 2 blocks. Each block stores the distances obtained when comparing the different map types used in this test (pairs of experimental cryo-EM maps and maps derived from atomic structures representing the same conformational state): AA (atomic versus atomic), AC (atomic versus cryo-EM), CA (cryo-EM versus atomic) and CC (cryo-EM versus cryo-EM). (Bottom) A consensus structure map for pairs of RyR1 conformations (from atomic model-derived simulated maps and from cryo-EM maps) resulting from the analysis of the blocks. The red circles are used to enhance the visualization of the different pairs. When compared with Fig. 4 ▸(*a*), it is possible to see that this decomposition of the distance matrix leads to a proper recovering of all the pairs found in the data set.

Our results, shown in Fig. 4 ▸, report the main difficulties that appear when mixing simulated and experimental cryo-EM maps. While the structure map based on *d*_1_ (the distance based on the amount of deformation) illustrates that many pairs are correctly placed together, the structure map based on *d*_2_ (the distance based on the similarity after undeforming) discriminated between maps derived from atomic models and maps coming from cryo-EM experiments. However, the point of this example was to intermix maps from different origins, so discrimination by origin was to be minimized, requiring a further adjustment to our approach. To tackle this problem, we extended our methodology by analysing separately the sub-blocks of the distance matrix including only atomic or only cryo-EM maps [see Fig. 5 ▸ (top)]. We thus performed the MDS of each one of the sub-blocks independently, obtaining the low-dimensional mappings *X*_AA_ and *X*_CC_ (the subscript indicates whether it corresponds to atomic/computational or cryo-EM/experimental maps). These two low-dimensional mappings were the input into the consensus procedure described in Section 2.4. Focusing on the consensus, we can see that the information provided by the two mappings *X*_AA_ and *X*_CC_ is combined into two different trajectories corresponding to each dimension in the distance matrices (simulated and experimental cryo-EM maps) that show a similar distance relationship among their points, illustrating that both trajectories correspond to the same states of RyR1. Therefore, the counterpart of each other, and their relative distances/positions, are retained [Fig. 5 ▸ (bottom)].

### 3.4. Experiment 4: application of the deformation field to atomic models of the CCT complex   

We described our deformation field **g**_*L*_ as a function that deforms *V*_1_ to let it become similar to *V*_2_, that is, as we have done in previous cases, acting only on two cryo-EM maps. However, since the deformation field is defined in the co­ordinate system of *V*_1_, it can also be applied to atomic models defined in the same coordinate system and not only to maps. In this way, we can also deform an atomic model defined for *V*_1_ and use it as starting point for a model of *V*_2_. Obviously, since the new atomic model defined in the coordinate system of *V*_2_ has been constructed purely based on geometrical considerations, all the stereochemical constraints have to be further imposed.

**Figure 6:**
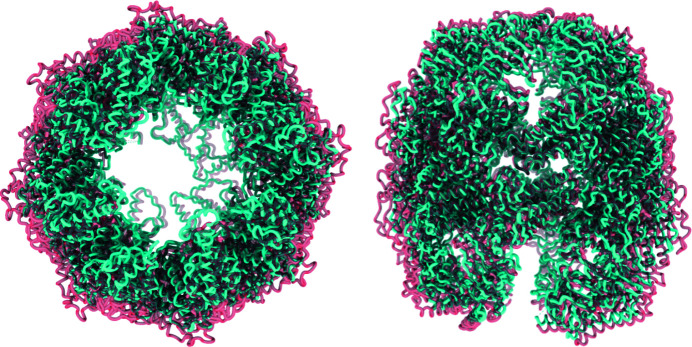
Deformation applied to one of the 30 CCT models obtained by the NMA-based approach called adaptive ANM described in Experiment 2. The deformation was computed using the cryo-EM maps simulated from the 30 models. The original atomic structure in the open state is shown in pink and the deformed version in the closed state in cyan. The results show that the deformation coefficients α_*l*, *n*, *m*_ computed with maps can be effectively applied to the atomic space of the model to approximate geometrically the conformation represented by the cryo-EM map at the level of atoms.

An example of an atomic model deformed following the previous procedure is presented in Fig. 6 ▸. The example was taken from the same data set as used in Experiment 2, which shows an open–closed transition of the CCT complex. The figure illustrates how the deformation applied to the atomic model of the open conformation results in an approximation to the closed conformation. Naturally, we can now compare this deformed model representing the closed conformation with the one obtained directly from the experimental map of the closed conformation. The r.m.s.d. (computed with *ChimeraX*) between these two models was 5.29 Å, certainly high, but substantially reduced compared with that between the open–closed models without applying any deformation, which was 7.90 Å. This r.m.s.d. reduction suggests that the deformation applied is appropriately reproducing a conformational change in the right direction, from open towards the closed state.

However, the overall scores obtained for the two deformed structures still show a high value, as many stereochemical features are not taken into account when computing the deformations. In order to improve the geometry of the deformed structures, we applied a real-space refinement [executing *Phenix* software (Liebschnerm *et al.*, 2019 ▸) with the default parameters] to the predicted structures using their respective electron-density maps. After this refinement, the r.m.s.d. value measured before decreased further to 4.52 Å. As a conclusion, the combination of deformation and refinement of atomic structures enables us to achieve predictions of different structure conformations on the path between two end points, suitable for performing further studies, though there is clearly room for improvement. For example, refining in between smaller deformations could be of benefit, *e.g.* in hybrid simulations methods where local refinement/simulation complements global deformations (Krieger *et al.*, 2020 ▸).

## 4. Conclusions   

The development of automatic algorithms to study continuous flexibility presented in this work results in simplified yet precise procedures, avoiding the need for user interference with the software and increasing the reproducibility of the results. It is also a significant step forward with respect to approaches like PCA and NMA of cryo-EM maps, avoiding the need to select components or modes and producing localized analysis.

The way this new approach works is by defining a new 3D basis where all deformation occurs. It is conceptually similar to the Fourier transform. The movements defining a transition between two different conformational states are decomposed into different components (that can be regarded as low-, medium- and high-frequency movements). Those components will depend on the degree of the basis used in the calculations. The displacements needed along each different component to minimize the distances between two electron-density maps are stored in a series of deformation coefficients **α**_*l*, *n*, *m*_, which can be further analysed to obtain the local strains and rotations undergone by the macromolecules during conformational transitions. The new approach thus unifies two of our previous developments (NMA and strain/rotation component extraction) for the analysis of continuous heterogeneity.

Apart from the information extracted from the deformation coefficients, our method allows for the definition of a distance measure based on the deformed electron-density maps, which is useful for building distance matrices. These distance matrices can be used afterwards to recover structure mappings that show the structural relationships existing among the diverse conformational states. Different definitions of the distance measures may focus on different aspects of the comparison. For this reason, we have devised a new procedure to combine several low-dimensional mappings into a single consensus mapping based on a minimum entropy criterion that tends to produce well ordered low-dimensional mappings and outperforms the results obtained by individual distance metrics.

The possibility of converting atomic models back to electron densities opens the possibility of a combined analysis on maps and models in the same conformational space. An illustrative example has been provided in Experiment 4, where cryo-EM maps, together with their respective structural models between two end points, have been represented in the same space as a set of experimental cryo-EM maps.

In the future, it may be interesting to explore alternative bases for the deformation field and the distance between volumes (like the Wasserstein distance).

## Supplementary Material

Video 1. DOI: 10.1107/S2052252521008903/eh5012sup1.mp4

Video 2. DOI: 10.1107/S2052252521008903/eh5012sup2.avi

## Figures and Tables

**Figure 7:**
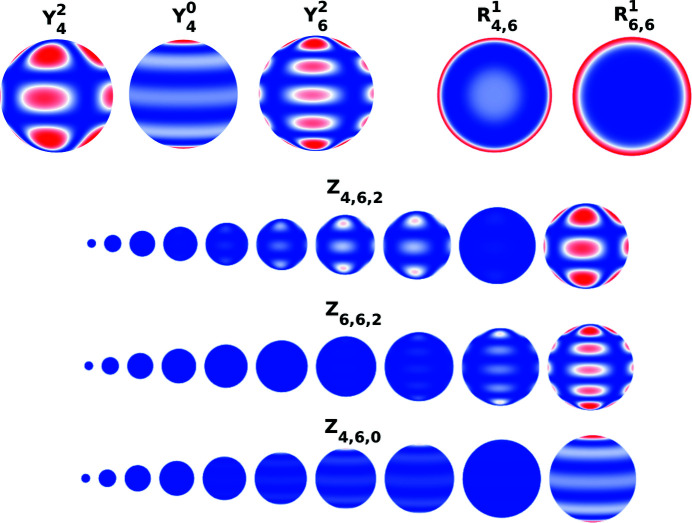
Representation of some components of the basis *Z*_*l*, *n*, *m*_ regarding their former angular and radial components 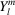 and 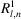 . Since the spherical harmonics 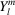 are only defined on the surface of the sphere, the representation of the basis components *Z*_*l*, *n*, *m*_ includes several spheres whose radius is contained in the interval [0, 1] to have a better graphical representation of the whole component. The real component would be obtained by stacking all the spheres (whose radii belong to the interval [0, 1]) concentrically. Each point in the three representations corresponds to the value obtained when evaluating the corresponding functions on a grid.

**Table 1:** List of real-valued spherical harmonics y_l^m ({\bf r}/|{\bf r}|)

	Order (*m*)						
Degree (*l*)	−3	−2	−1	0	1	2	3
0				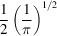			
1			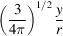	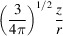	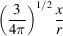		
2		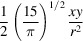	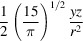	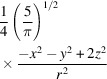	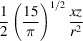	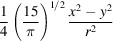	
3	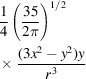	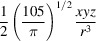	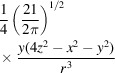	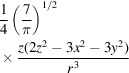	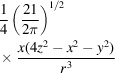	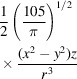	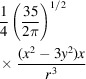

**Table 2:** Generalized and normalized radial Zernike polynomials

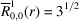	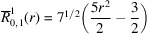	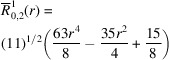	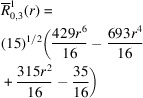	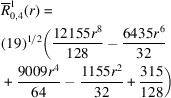
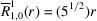	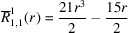	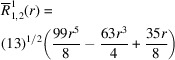	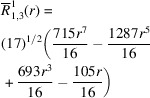	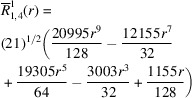
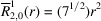	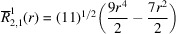	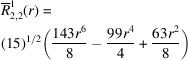	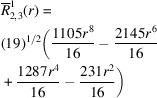	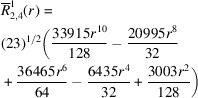
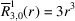	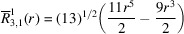	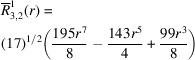	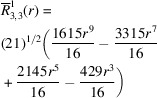	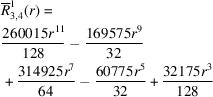
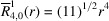	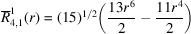	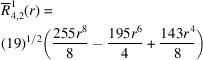	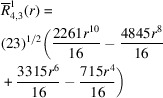	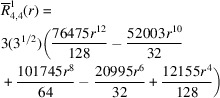
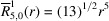	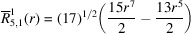	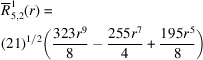	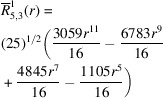	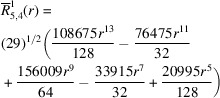

## Appendix A 3D real-valued generalized Zernike polynomials

In this section we discuss the functions that we use as basis functions of the deformations in the unit ball *B*. We use the generalized Zernike polynomials defined on the 3D ball; in Appendix *B*1, we briefly review the relation of this basis to the better-known 2D form of Zernike polynomials.

In general, the expansion of any real valued function *g*(**r**) ∈ *L*_2_(*B*) in this basis is defined by the formula

where α_*l*, *n*, *m*_ are real-valued coefficients, and *Z*_*l*, *n*, *m*_(**r**) are the 3D real-valued (normalized) generalized Zernike polynomials defined by the formula

where *r* is the radial component of the 3D coordinate **r**, θ and ϕ are its polar and azimuthal angles, respectively, in spherical coordinates, *n* and *l* are non-negative integers, and *m* is an integer such that −*l* ≤ *m* ≤ *l*. We refer to *l* as the *spherical frequency*.  is the real-valued spherical harmonic defined by the formula

where  are the associated Legendre polynomials defined by the formula

The real and imaginary parts of the complex-valued spherical harmonics are available in standard textbooks such as that by Abramowitz & Stegun (1966 ▸). For completeness, Table 1 ▸ shows these spherical harmonics in Cartesian coordinates. Before defining the normalized generalized radial Zernike polynomials denoted by  above, we define the (un­normal­ized) generalized radial Zernike polynomials  as follows:

where  are the Jacobi polynomials,

The definitions and properties of the standard associated Legendre polynomials and Jacobi polynomials are available, *inter alia*, in the book by Abramowitz & Stegun (1966 ▸).

Finally, while the radial polynomials are orthogonal (with the appropriate norm), they are not orthonormal. This is easily corrected by replacing the radial polynomials with the normalized generalized radial Zernike polynomials, denoted by  ,

The parameter *p* is associated with the choice of inner product and the dimensionality of the balls; in the case of a 3D ball, the natural choice of *p* is *p* = 1, which yields the basis in (16) which is orthonormal in the natural inner product on *L*_2_(*B*). We give in Table 2 ▸ the explicit list of radial functions  that we use.

We recognize that our choice of basis functions for the expansion is certainly not the only possible choice. We used the Zernike polynomials (in the generalized form presented here) to obtain an expansion of functions in a ball which do not vanish at the boundaries. Our use of Zernike polynomials also yields a basis that is closed under rotations. The graphical representation of some components of the basis is shown in Fig. 7 ▸.

## Appendix B Properties and relationships of this basis

### b1. Properties of the polynomials involved – Zernike polynomials

We note that slightly different definitions and normalization are used in different sources; the most commonly used form of Zernike polynomials is associated with 2D functions on the unit disc, whereas we are interested in 3D functions on the unit ball. The better-known traditional radial Zernike polynomials, denoted here by  , are a special case of the generalized radial Zernike polynomials  ,

with  if *l* − *m* is odd or if *m* > l. The definition of the 3D real-valued Zernike polynomial [equation (16)] is analogous to the definition of the traditional 2D Zernike polynomials  on the unit disc,

Unfortunately, the common notation for the 2D and 3D cases can be misleading: the parameter *m* here plays the role of the parameter *l* in the analogous 3D case; the parameter *m* in the 3D case is related to the existence of both sine and cosine for each *m* here, but does not otherwise have an immediate counterpart here.

Some properties of these generalized Zernike polynomials, including the higher-dimensional cases, are discussed in further detail by Slepian (1964 ▸), Serkh (2015 ▸), Greengard & Serkh (2018 ▸) and Lederman (2017 ▸).

The radial Zernike polynomials in equation (19) are orthogonal with respect to the inner product,

so that  if *n*_1_ ≠ *n*_2_. Note that they are not necessarily orthogonal for different *l*, that is,  is not 0, in general. It follows that the Zernike polynomials *Z*_*l*, *n*, *m*_ (16) are orthogonal (across all different combinations of *n*, *l* and *m*) on the natural inner product on the unit ball.

### b2. A remark on numerical evaluation

As is the case with many orthogonal polynomials, the direct computation using the explicit sum of monomials is generally unstable and not recommended in numerical computation. However, in this work, since we truncate the polynomials at low *n*, the explicit form has been found experimentally to be sufficiently stable. For additional details on computation see Lederman (2017 ▸).

### b3. Closure under rotations

We recall that we use basis functions defined in equation (15), which are composed of a radial component and an angular component. Furthermore, we truncate the expansion in equation (2) such that if *Z*_*l*, *n*, *m*_(**r**) is in the expansion, then  is also in the expansion for any −*l* ≤ *m*, *m*′ ≤ *m*. As is well known, the rotation of the frame of reference of spherical harmonics of a given spatial frequency *l* is a unitary operation and closed rotations. If follows that the linear combination of  is closed under rotations. In other words, regardless of the frame of axis we choose for our spherical harmonics, we can represent the same functions using our choice of basis.

We recall that the deformation field **g**_*L*_(**r**) is a 3D vector defined in equation (2) at any point *r*. If we rotate the axes *x*, *y* and *z* we must rotate the vector **g**_*L*_(**r**) to obtain a vector in the new coordinate system. As is well known,

where *A* is the appropriate unitary rotation matrix.

It follows that the basis we have chosen is closed under rotations; regardless of the orientation of the frame of reference we choose (but depending on the position of the centre), we can represent the same fields. Furthermore, the transformation between frames of reference is unitary. We note that, since the volume is defined on the grid, the problem definition is not entirely closed under rotations, although the definition of the field is closed under rotations.

## Appendix C Complex-valued Zernike 3D basis

We may extend our basis function to a complex-valued Zernike 3D basis. This second basis uses spherical harmonics, which are well known basis functions for functions defined over the surface of a unit sphere. For doing so, we should define  [see equation (16)],

where  are the standard spherical harmonics,

It is well known that the spherical harmonics are a complete orthonormal basis on the surface of the unit sphere such that

where Ω is the surface of the unit sphere.

In this new basis the expansion would be expressed as

The relationship between the expansion that uses the real-valued basis functions and the expansion that uses the complex-valued basis functions is

and for the basis functions

Using the fact that the radial Zernike polynomials are a complete radial orthonormal basis, and the fact that spherical harmonics are a complete orthonomal basis on the the surface of a sphere, one can show that the generalized Zernike polynomials are a complete orthonormal basis of function on the unit ball with respect to the natural *L* ^2^ norm on the unit ball.

Suppose that **r** is a 3D coordinate of a point in real space and **R** is a 3D frequency. Then the Fourier transform of the complex-valued basis function would be

where 〈**r**, **R**〉 is the usual inner product in  , *R* is the modulus of **R** and *J*_α_(*x*) is the Bessel function of the first kind and order α.
